# The IgV domain of the poliovirus receptor alone is immunosuppressive and binds to its receptors with comparable affinity

**DOI:** 10.1038/s41598-023-30999-w

**Published:** 2023-03-21

**Authors:** Shrayasee Saha, Amanda Sparkes, Esther I. Matus, Peter Lee, Jean Gariépy

**Affiliations:** 1 Physical Sciences, Sunnybrook Research Institute, Toronto, Canada; 2 Department of Pharmaceutical Sciences, University of Toronto, Toronto, Canada; 3 Department of Medical Biophysics, University of Toronto, Toronto, Canada; 4 Physical Sciences, Sunnybrook Research Institute, 2075 Bayview Ave., Room M7-434, Toronto, ON M4N 3M5 Canada

**Keywords:** Chronic inflammation, Recombinant protein therapy

## Abstract

PVR (poliovirus receptor) functions as a ligand that signals through TIGIT and CD96 to induce suppression of T-cell and NK-cell responses. Alternatively, PVR binds to CD226, resulting in a co-stimulatory signal. To date, TIGIT antibody antagonists have been developed to restore immune functions and allow PVR to signal though CD226 in the context of cancer immunotherapy. Due to PVR receptor heterogeneity, agonizing either of these pathways with a recombinant form of the PVR extracellular domain represents a therapeutic strategy for either immunosuppression or activation. Here, we developed a minimal murine PVR-Fc fusion construct, consisting of only the IgV domain of PVR (vdPVR-Fc), and assessed its ability to dampen inflammatory responses in a murine model of psoriasis. vdPVR-Fc and PVR-Fc containing the full-length extracellular domain bound to TIGIT, CD96 and CD226 with similar low nanomolar affinities as defined by surface plasmon resonance. vdPVR-Fc was also able to suppress the in-vitro proliferation of murine CD4^+^ and CD8^+^ T-cells in mixed splenocyte cultures. Importantly, vdPVR-Fc delayed the onset, and reduced inflammatory responses (scaling and thickness) in a murine model of psoriasis. Collectively, our results suggest that the minimal IgV domain of PVR is sufficient to dampen immune responses in-vitro and attenuate symptoms of psoriasis in-vivo.

## Introduction

T-cells express a wide range of co-inhibitory and co-stimulatory receptors that tightly regulate immune responses against invading pathogens or malignant cells, while maintaining self-tolerance. The antibody-mediated blockade of co-inhibitory receptors PD-1 and CTLA-4 has been shown to clinically improve anti-tumor immune responses in multiple human malignancies^1^. In contrast, agonising these co-inhibitory receptors can be exploited to treat autoimmune diseases and/or suppress inflammation^2,3^.

Engineering natural ligands to agonize receptors represents a valid therapeutic strategy. One example of this strategy is the use of Galectin 9 to signal through the inhibitory TIM3 receptor to dampen inflammatory responses in the context of mouse models of experimental autoimmune encephalomyelitis (EAE), multiple sclerosis and collagen-induced arthritis^3^. Fusing natural ligands to an Fc domain, can also increase their avidity towards their receptors, their serum half-lives and allow for more robust in-vivo crosslinking events^4^.

The extracellular domain (ECD) of the polio virus receptor (PVR; CD155), expressed on antigen presenting cells and tumor cells, is composed of an N-terminal IgV region followed by two IgC elements. This ECD has been shown to bind to T-cell immunoglobulin and ITIM domain (TIGIT; also known as WUCAM. VSTM3 and VSIG9), a co-inhibitory receptor that is expressed on activated CD4^+^ T-cells, CD8^+^ T-cells and NK-cells, where it suppresses their activity^5,6^. Furthermore, PVR binds to the co-inhibitory receptor CD96 (TACTILE) and the costimulatory receptor CD226 (DNAM1) which are also expressed on T-cells and NK-cells^7,8^. Interestingly, TIGIT inhibits CD226-mediated co-stimulation by blocking CD226 dimerization^9^. TIGIT has also been reported to bind to PVR with high affinity relative to CD226^10^. Finally, CD96 has been demonstrated to compete for PVR binding to CD226 to directly inhibit NK-cell functions^7^. Consequently, unlike receptor-specific mAbs, engineering PVR ECD constructs would simultaneously target both TIGIT and CD96 inhibitory pathways, representing a biologic with a potentially broader anti-inflammatory property. Engagement of the TIGIT inhibitory pathway has been previously reported to reduce the severity of EAE and delay the development of systemic lupus erythematosus^11,12^.

Here, we describe the generation of a PVR-Fc fusion construct consisting of only the smallest functional IgV domain (vdPVR-Fc). We demonstrate that this minimal domain of PVR as an Fc construct binds to TIGIT, CD226 and CD96 with similar nanomolar K_d_s. Finally, vdPVR-Fc displays immunosuppressive activities in the context of inhibiting T-cell proliferation in-vitro and its ability to attenuate the development of psoriasiform-like symptoms in a murine model of psoriasis.

## Results

### Design and production of murine vdPVR-Fc and mutant vdPVR-Fc

**Figure 1:**
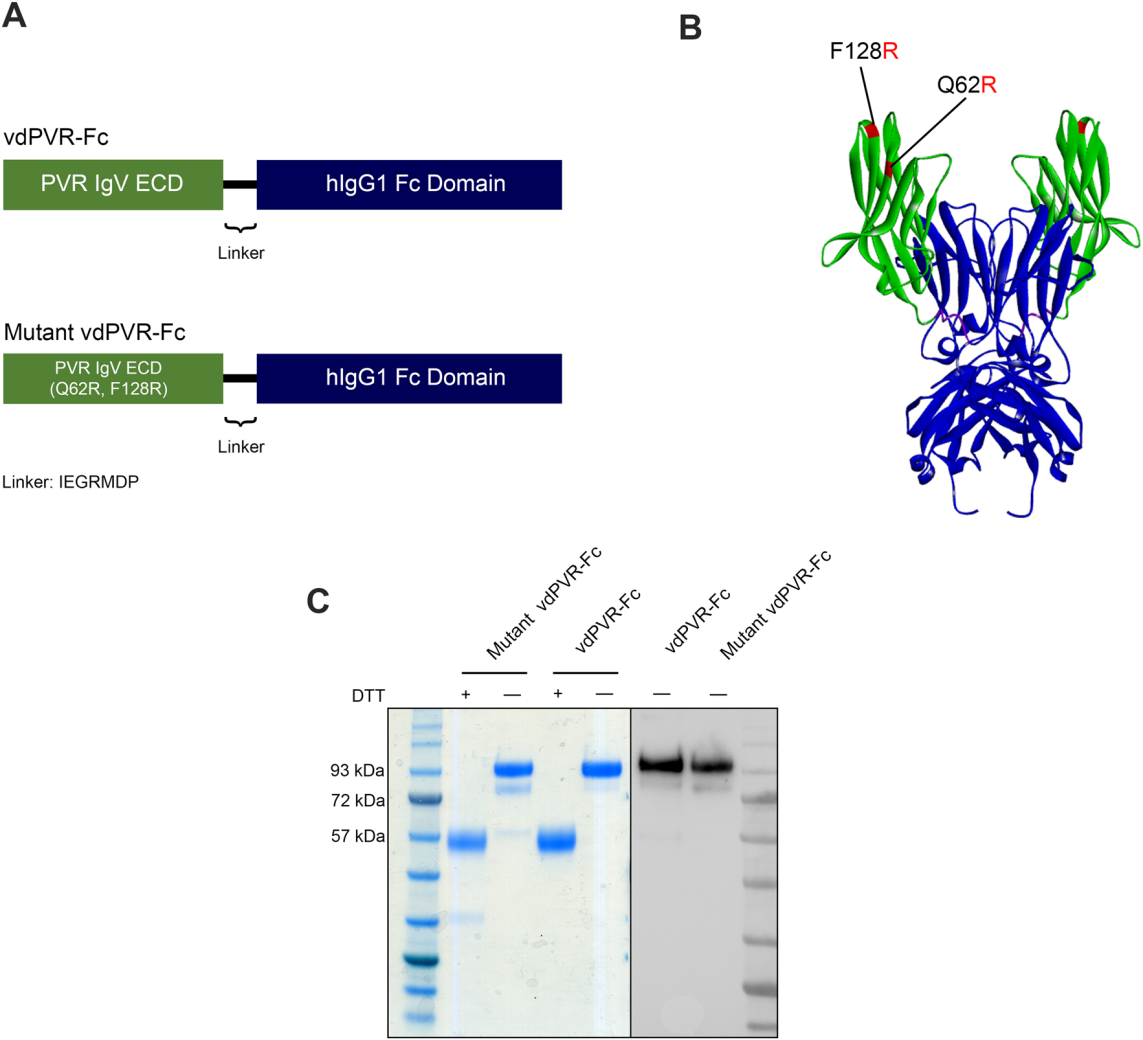
Design and purification of murine vdPVR-Fc and mutant vdPVR-Fc. **(A)** Diagram depicting the structure of IgV-domain PVR-Fc (vdPVR-Fc) and mutant vdPVR-Fc. The vdPVR-Fc recombinant construct consists of the N-terminal murine IgV portion of the PVR extracellular domain (residues 29–147; Accession # NP_081790.1) connected by a short linker (IEGRMDP) to the human IgG1 Fc domain (residues 100–330; Accession # P01857) located at its C-terminus. Mutant vdPVR-Fc contains two point mutations in the PVR IgV domain (Q62R and F128R). **(B)** Ribbon structure representation of the mutant vdPVR-Fc construct generated using ColabFold. Color pattern: vdPVR (in green), linker (in purple) and Fc domain (in blue). Q62R and F128R mutations in vdPVR are highlighted in red. **(C)** The purity and molecular mass of both proteins were confirmed by SDS-PAGE (left panel) in the presence or absence of a reducing agent (DTT). Western blot (right panel) of both constructs in the absence of DTT (blot detected using an anti-human Fc fragment antibody conjugated to HRP). Both expressed proteins migrated as a 94 kDa band in their dimeric form.

The recombinant PVR-Fc fusion protein (vdPVR-Fc) was created by fusing the IgV domain of murine PVR ECD to a human IgG1 Fc domain (Fig. 1A). A similar construct was designed to incorporate two amino acid mutations (Q62R and F128R; highlighted in red) (Fig. 1B), previously shown to abrogate PVR binding to TIGIT and CD226^13,14^. This construct, termed mutant vdPVR-Fc, served as a negative control for this study. Both constructs were expressed as secreted soluble proteins in Expi293 mammalian cells and purified using Protein A affinity chromatography. The purity and molecular weight of the proteins were confirmed by SDS-PAGE and Western Blot analysis. As shown in Fig. 1C, both proteins migrated at the expected molecular weight for the dimeric form of vdPVR-Fc (~ 94 kDa) in the absence of a reducing agent (Fig. 1C).

### vdPVR-Fc binds with high affinity to TIGIT, CD226 and CD96

**Figure 2:**
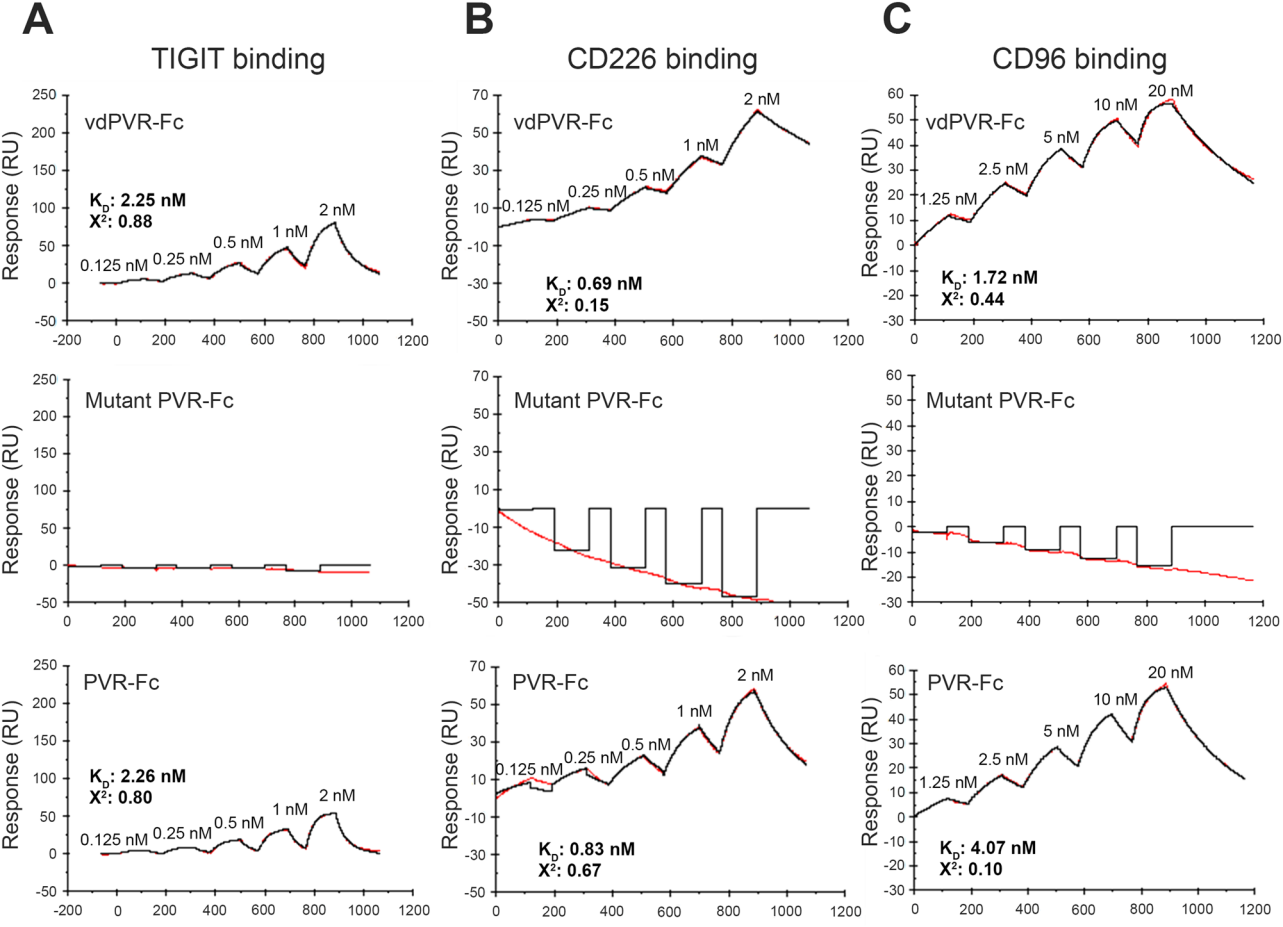
Surface plasmon resonance (SPR) titration profiles of vdPVR-Fc, mutant vdPVR-Fc and PVR-Fc binding to mouse TIGIT, mouse CD226 and mouse CD96. **(A-C)** SPR single-cycle kinetics sensorgrams (in red) and fitted curves (in black) with equilibrium association (K_D_) constant showing the binding of vdPVR-Fc, mutant vdPVR-Fc and full length PVR-Fc to immobilized recombinant **(A)** mouse TIGIT-Fc-his, **(B)** mouse CD226-his and **(C)** CD96-his. Five concentrations (indicated on the sensorgrams) of vdPVR-Fc, mutant vdPVR-Fc and PVR-Fc were flown over the immobilized recombinant proteins. Kinetic parameters were calculated from 1:1 Langmuir binding model using Biacore evaluation software.

The binding of vdPVR-Fc and mutant vdPVR-Fc to recombinant mouse TIGIT, CD226 and CD96 was assessed using surface plasmon resonance (SPR). vdPVR-Fc was shown to bind to TIGIT-Fc-his, CD96-his, and CD226-his with similar nanomolar affinities (K_d_ values of 2.25 nM, 1.72 nM and 0.69 nM respectively) (Fig. 2A–C, Table 1). We compared these results to binding constants obtained using full length PVR-Fc to determine whether our modifications to the ECD of PVR impacted its ability to bind to its cognate receptors. Full length PVR-Fc bound to all three cognate receptors with similar low nanomolar affinities (TIGIT-Fc, K_d_ 2.26 nM; CD96-his, K_d_ 4.07 nM, CD226-his, K_d_ 0.83 nM) (Fig. 2A–C, Table 1) as was obtained for vdPVR-Fc. Finally, we assessed whether the mutations introduced in the IgV domain of PVR nullified its ability to bind to its cognate receptors by comparing the affinities obtained to those of vdPVR-Fc or the full-length mouse PVR ECD-Fc. As expected, the negative control, mutant vdPVR-Fc, did not bind to either mouse TIGIT-Fc, CD226-his or CD96-his as indicated by the lack of increase in response units from the SPR sensorgrams (Fig. 2A–C).

**Table 1:** Summary of vdPVR-Fc and PVR Fc binding kinetic parameters calculated from SPR sensorgrams.

Interaction	k_on_ (1/Ms)	k_off_ (1/s)	K_D_ (M)	Chi^2^
vdPVR-Fc and mTIGIT	2.92 × 10^7^	6.56 × 10^2^	2.25 × 10^–9^	0.88
vdPVR-Fc and mCD226	2.67 × 10^6^	1.84 × 10^–3^	6.90 × 10^–10^	0.15
vdPVR-Fc and mCD96	3.52 × 10^6^	2.86 × 10^–3^	1.72 × 10^–9^	0.44
PVR-Fc and mTIGIT	2.25 × 10^7^	6.46 × 10^2^	2.26 × 10^–9^	0.80
PVR-Fc and mCD226	3.20 × 10^6^	6.51 × 10^–3^	8.30 × 10^–10^	0.67
PVR-Fc and mCD96	1.17 × 10^6^	4.55 × 10^–3^	4.07 × 10^–9^	0.10

### vdPVR-Fc binds to the surface of murine T-cells

**Figure 3:**
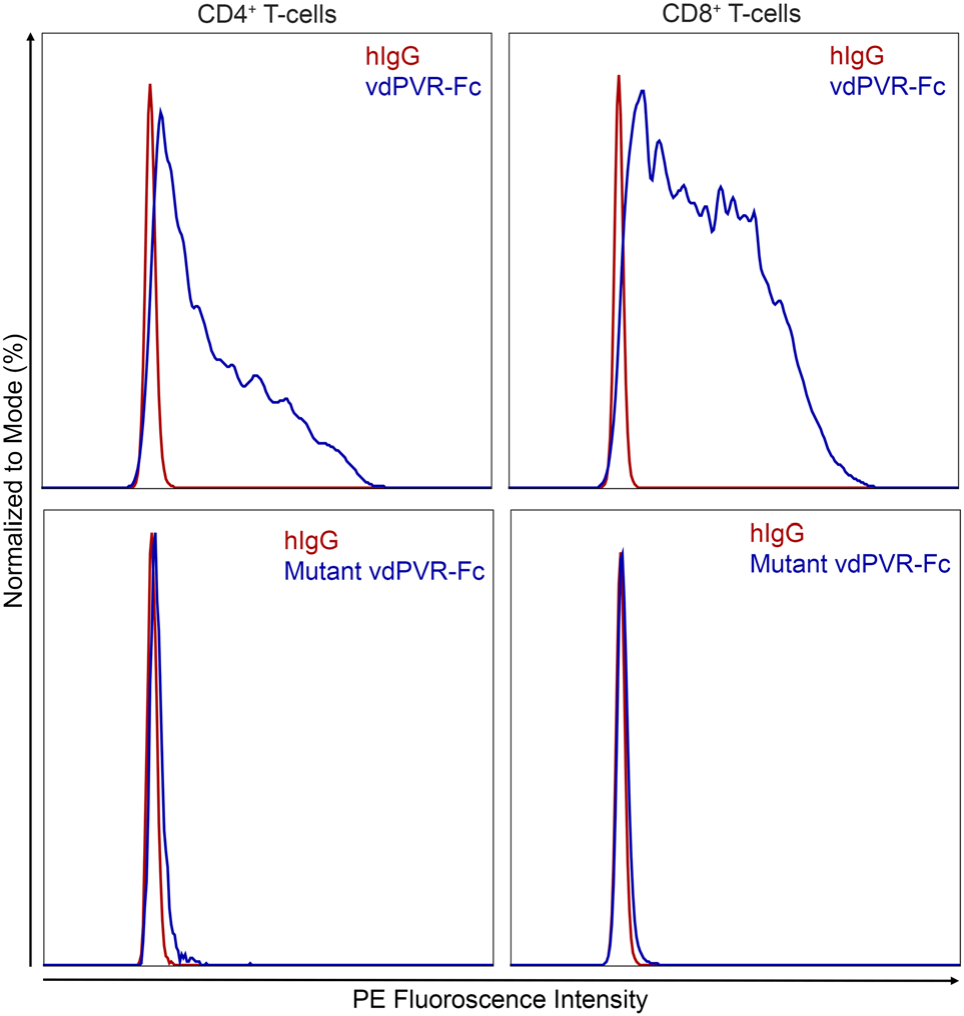
vdPVR-Fc binds to primary mouse T-cells. Stimulated T-cells in mixed splenocyte cultures were incubated with 2.5 µg of either vdPVR-Fc and mutant vdPVR-Fc. Histograms depicting vdPVR-Fc (blue) and mutant vdPVR-Fc (blue) binding to CD4^+^ and CD8^+^ T-cells compared with a human IgG1isotype control (red). Binding of vdPVR-Fc and mutant vdPVR-Fc to T-cells was detected with an anti-human IgG1 Fc antibody conjugated to PE. Data is representative of 2 independent experiments.

To determine if vdPVR-Fc can bind to the surface of intact cells, we assessed its ability to bind to the surface of stimulated T-cells in a mixed splenocyte culture. Herein, PE-conjugated anti human IgG1-Fc was used to detect vdPVR-Fc binding to murine T-cells by flow cytometry. As shown in Fig. 3, vdPVR-Fc bound to both CD4^+^ and CD8^+^ T-cell subsets as evidenced by the large shift in the PE signal intensity relative to the human IgG1 isotype. In contrast, mutant vdPVR-Fc did not bind to either T-cell subsets (Fig. 3).

### vdPVR-Fc suppresses murine T-cell activity in-vitro

**Figure 4:**
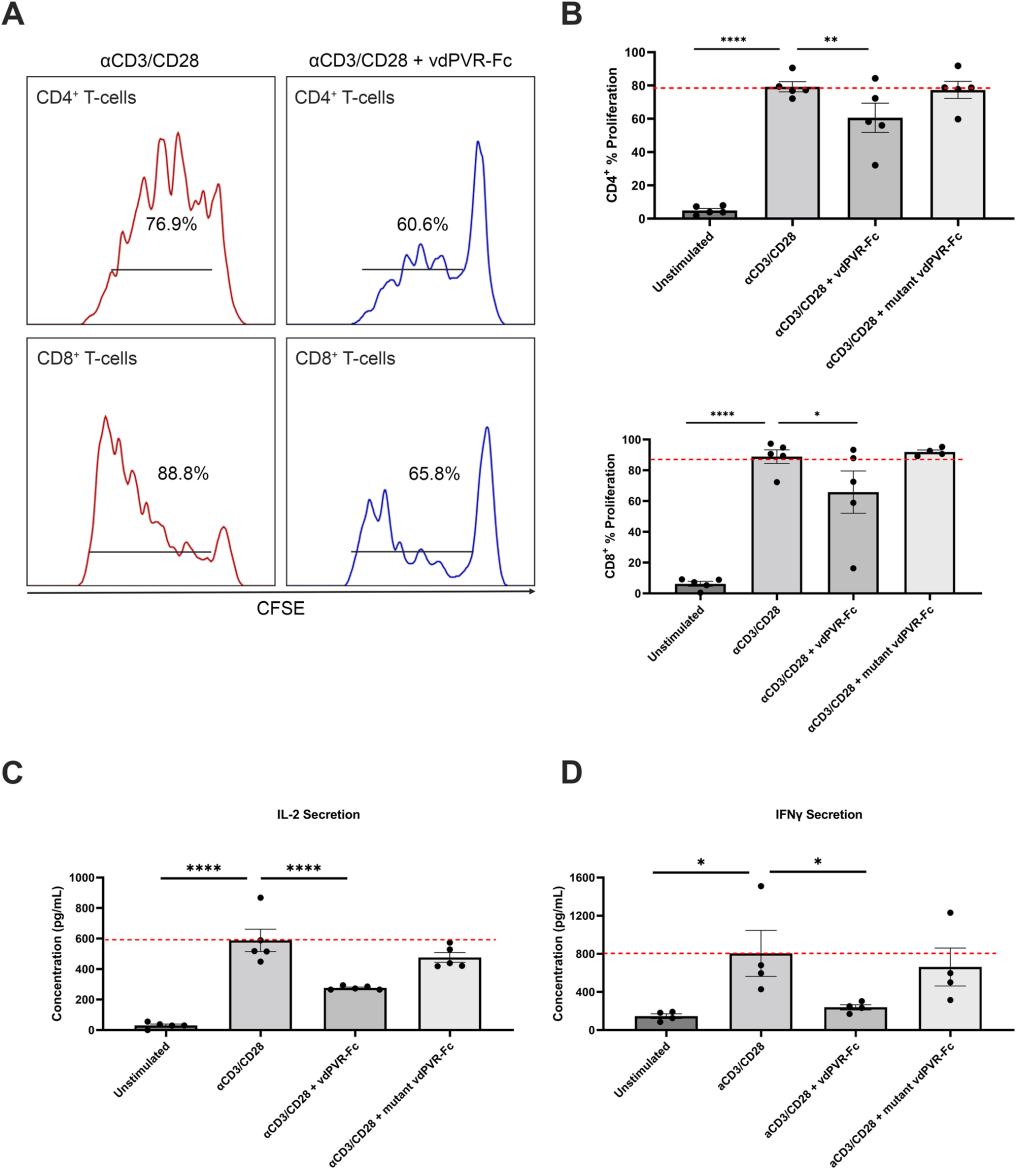
vdPVR-Fc as a ligand suppresses T-cell activity in mouse splenocyte cultures in-vitro. **(A)** Representative CFSE profiles showing the proliferation percentages of murine T-cells in splenocyte cultures stimulated with anti-CD3/CD28 beads in the presence or absence of vdPVR-Fc (10 µg/mL) for 3 days. **(B)** Graph showing the mean proliferation percentages of CD4^+^ and CD8^+^ T-cells unstimulated, stimulated with anti-CD3/CD28 coated beads, or with anti-CD3/CD28 beads in the presence of either vdPVR-Fc (10 µg/mL) or mutant vdPVR-Fc (10 µg/mL) after 3 days of culture. Proliferation was measured by flow cytometry as a measure of the percentage of CFSE signal associated with cell divisions over 3 days. The culture supernatants were harvested from the murine splenocyte culture after 3 days and the level of **(C)** IL-2 and **(D)** IFNγ were quantified by ELISA. Red line represents maximum stimulation using anti-CD3/CD28 coated beads. Each point on the graph represents a biological replicate. Error bars represent mean ± SEM, *p < 0.05, **p < 0.01 and ****p < 0.0001 evaluated by one-way ANOVA relative to anti-CD3/CD28 coated beads alone.

**Figure 5:**
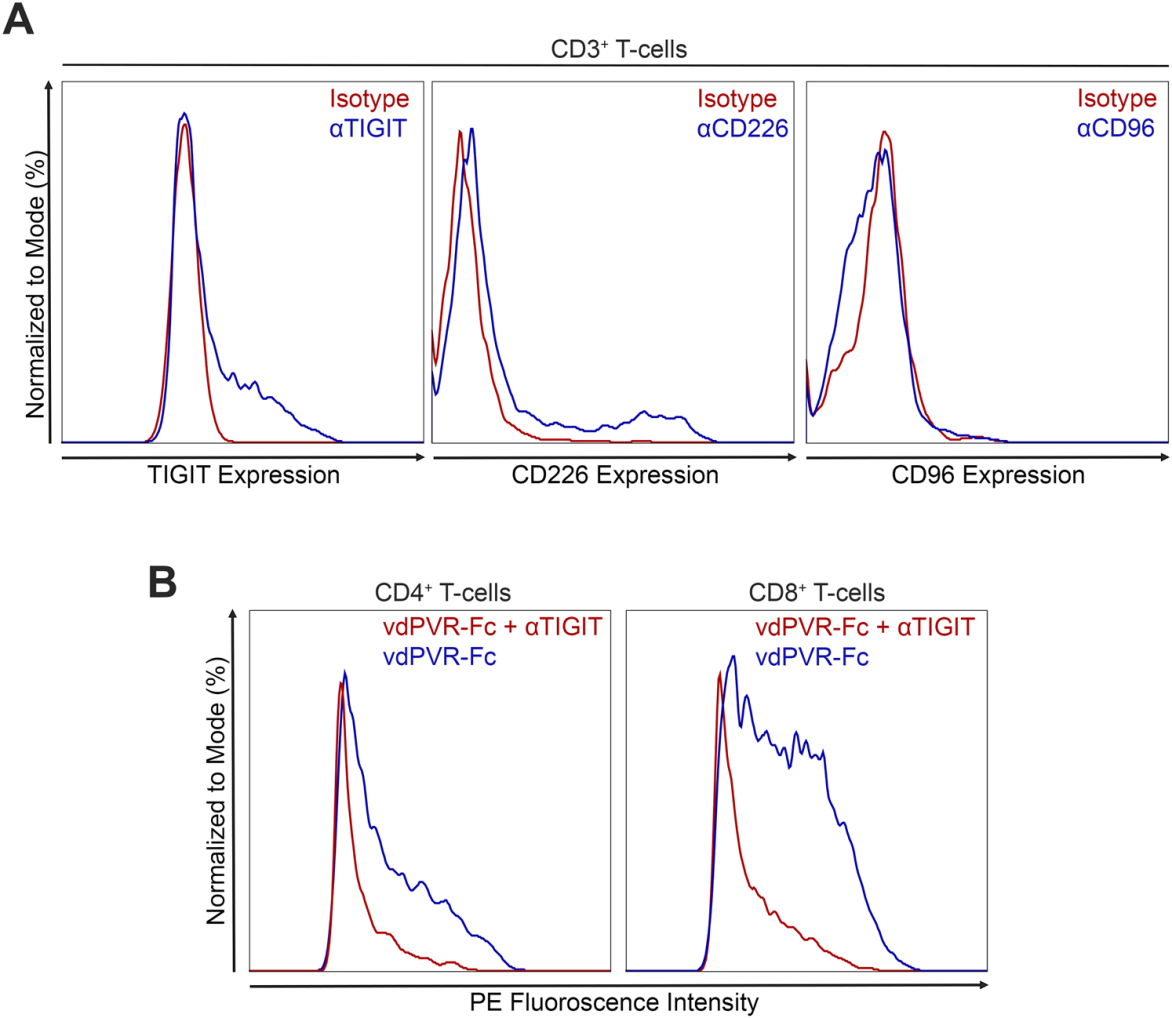
Expression of PVR cognate receptors on T-cells and cell binding competition. **(A)** TIGIT, CD226 and CD96 expression on CD3^+^ T-cells was assessed after 1 day of stimulation with anti-CD3/CD28 beads. **(B)** Flow cytometry histograms showing vdPVR-Fc binding to stimulated CD4^+^ and CD8^+^ T-cell subsets (blue) and vdPVR-Fc binding after the addition of 2-molar excess anti-TIGIT antibody (red). Data are representative of 2 independent experiments.

The functional ability of vdPVR-Fc to suppress T-cell activity in-vitro was tested using anti-CD3/CD28-stimulated murine splenocytes in the presence or absence of vdPVR-Fc or mutant vdPVR-Fc (Fig. 4A,B). T-cell proliferation responses as well as IL-2 and IFNγ cytokine secretion levels were assessed (Fig. 4B–D). CFSE histograms of T-cells indicate that the addition of vdPVR-Fc was able to significantly inhibit the proliferation of anti CD3/CD28 stimulated CD4^+^ T-cells (from 76.9% down to 60.6% proliferation; p = 0.0062) and CD8^+^ T-cells (from 88.8% down to 65.8% proliferation; p = 0.0390) as compared to anti CD3/CD28 stimulation alone (Fig. 4A,B). Mutant vdPVR-Fc showed no considerable changes in either CD4^+^ or CD8^+^ T-cell proliferation compared to anti CD3/CD28 stimulation alone (Fig. 4B). Furthermore, vdPVR-Fc significantly reduced the level of both IL-2 (p < 0.0001) and IFNγ (p = 0.0459) secretion in the splenocyte culture as compared to anti-CD3/CD28 alone (Fig. 4C,D). As expected, mutant vdPVR-Fc had negligible effects on both IL-2 and IFNγ production (Fig. 4C,D). Despite CD226 being expressed on the surface of the T-cells (Fig. 5A), a net immunosuppressive effect was observed. To verify that vdPVR-Fc is binding to TIGIT and/or CD96, explaining this suppressive effect, we verified that TIGIT was expressed on the surface of CD3^+^ T-cells stimulated in-vitro (Fig. 5A). We were unable to detect the expression of CD96 on CD3^+^ T-cells indicating that the suppression is likely mediated by TIGIT. These results indicate that the observed effects are not due to Fc-mediated toxicity as confirmed by assaying for the presence of lactate dehydrogenase (LDH) in the culture supernatant (Supplementary Fig. 1). None of the compounds tested were identified as being cytotoxic under these culture conditions. To further assess the specificity of vdPVR-Fc for TIGIT, an anti-murine TIGIT antibody was used to compete for the binding of vdPVR-Fc on maximally stimulated T-cells (to ensure TIGIT expression). Specifically, the addition of a 2-molar excess of an anti-TIGIT antibody, known to block the PVR/TIGIT interaction^11^, was able to displace vdPVR-Fc  bound to the surface of both T-cell subsets (Fig. 5B).

### vdPVR-Fc delays the onset of psoriasiform symptoms in mice

**Figure 6:**
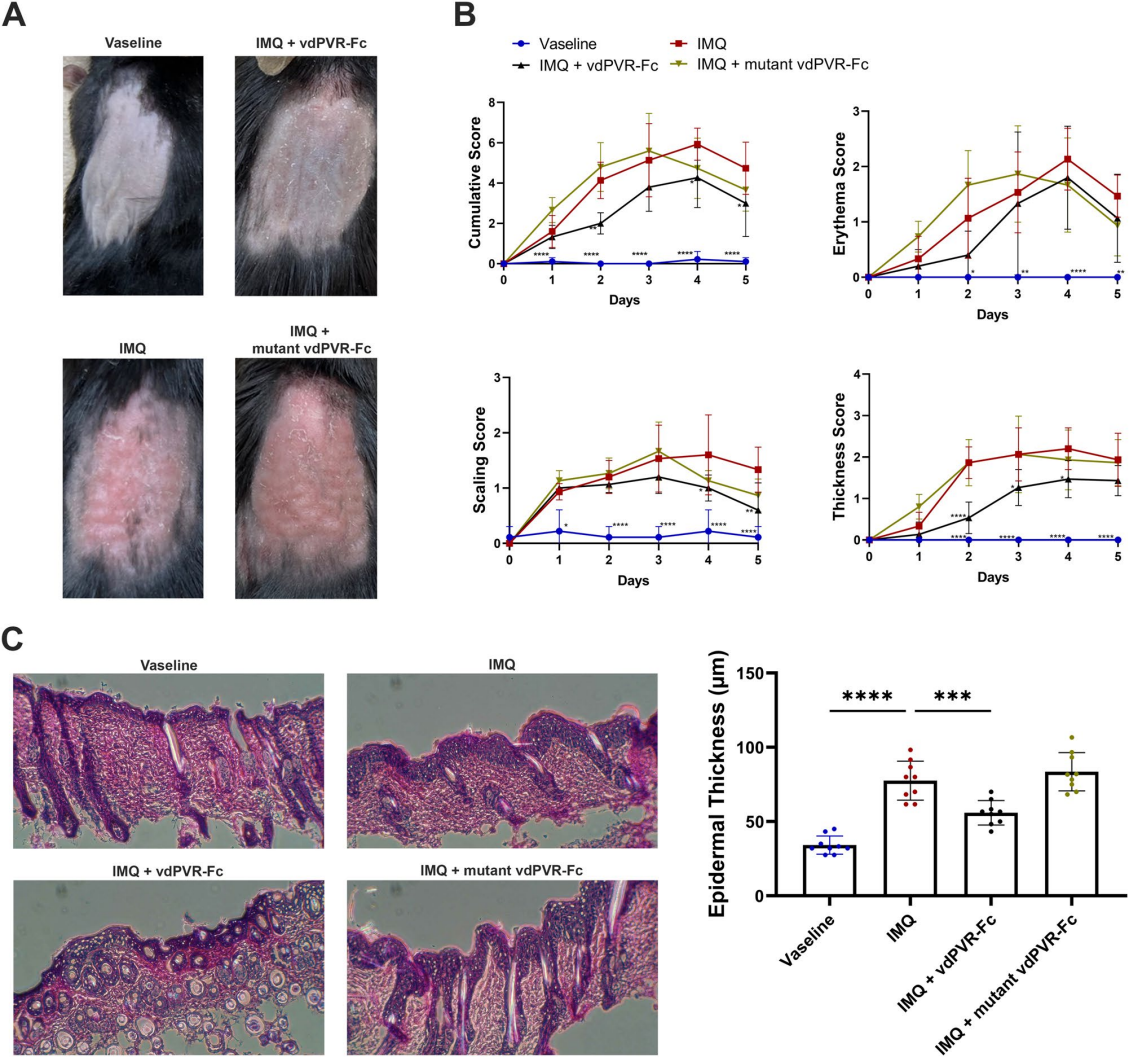
vdPVR-Fc delays the onset of psoriatic symptoms in a murine model of imiquimod-induced psoriasis. Female C57BL/6 mice received a daily topical application of either Vaseline or 5% imiquimod (IMQ) cream on their shaved backs for 5 days. IMQ-treated mice received intraperitoneal  doses of either vdPVR-Fc or mutant vdPVR-Fc every other day starting from Day 0. **(A)** Representative images of mouse back skin on Day 2 after treatment with either Vaseline, imiquimod alone, imiquimod and vdPVR-Fc or imiquimod and mutant vdPVR-Fc. **(B)** Individual (erythema, scaling, and thickness) and cumulative scores of the treatment groups (n = 5). Graphs show an average score of 3 independent scorers comparing each group to IMQ alone (adjusted p value, evaluated by two-way ANOVA). **(C)** Hematoxylin and eosin staining of back skin sections of Vaseline, imiquimod alone, vdPVR-Fc and mutant vdPVR-Fc treated mice. Back skin samples were collected on Day 3. Epidermal thickness was quantified from histology sections (n = 9). Error bars represent mean ± SD, *p < 0.05, **p < 0.01 and ****p < 0.0001 relative to IMQ alone group evaluated by ANOVA.

PVR-Fc has not been tested in the context of an in-vivo mouse model of psoriasis. In humans, TIGIT expression is correlated with psoriasis severity^15,16^. Furthermore, CD4^+^ T-cells isolated from patients treated with human PVR-Fc ex-vivo show significant reduction in proliferation and pro-inflammatory cytokine production^15^. Herein, we assessed the effect of treating mice displaying Imiquimod (IMQ)-induced psoriatic lesions with vdPVR-Fc. As shown in Fig. 6A, there is a marked improvement in the disease severity early in the study in the mice treated intraperitoneally with vdPVR-Fc as compared to IMQ alone or the mutant vdPVR-Fc. Daily quantification of the symptom severity (erythema, scaling and thickness) (Fig. 6B) indicated a delayed onset of psoriatic symptoms and minimized peak severity (Day 3–4). The delayed onset of disease was particularly evident based on the reduced thickness and scaling of the epidermis observed in vdPVR-Fc-treated mice, as compared to mice receiving IMQ alone or for IMQ-treated mice given mutant vdPVR-Fc. These results were further supported by the subsequent histological assessment of skin sections (Fig. 6C); mice treated with vdPVR-Fc displayed significantly lower epidermal thickness, comparable to Vaseline (vehicle)-treated mice, relative to mice given IMQ alone (p = 0.0004). These results may be attributed, at least in part, to a significant reduction in CD11b^+^ NKp46^+^ NK cells (p = 0.0304) within the skin (Supplementary Fig. 2). There were no notable differences in any of the other cell populations examined at peak severity (CD11b^+^ F4/80^+^ macrophages, CD11b^+^ CD11c^+^ dendritic cells, CD11b^+^ Ly6C^++^ monocytes, CD4^+^ T-cells and CD8^+^ T-cells (data not shown)). Based on these results, vdPVR-Fc is able to reduce the proliferation of T-cells and secretion of pro-inflammatory cytokines in-vitro, as well as attenuate psoriasiform-like symptoms in an in-vivo mouse model of psoriasis.

## Discussion

The PVR/TIGIT/CD226/CD96 signalling axis represents a promising therapeutic target for both cancer immunotherapy and the treatment of autoimmune diseases. PVR binds to TIGIT and CD96 to suppress T-cell and NK cell functions^5–7^. In contrast, PVR engagement of CD226 results in co-stimulatory signalling^8^. Accordingly, CD226 agonism in combination with appropriate antagonistic anti-TIGIT and/or anti-CD96 antibodies represents an attractive therapeutic strategy in the context of cancer immunotherapy. To date, attempts to modulate these immunoregulatory pathways have been centered around TIGIT antagonism and/or CD226 agonism in the context of cancer immunotherapy utilizing antibodies, with mixed results being observed in clinical trials^17,18^. The use of natural ligands to agonize immunoregulatory pathways is largely underscored partly due to receptor heterogeneity (i.e. the ligand binds to multiple receptors with opposing outcomes)^19^. While receptor heterogeneity holds true for PVR, the interplay between the receptors i.e. TIGIT blocking CD226 mediated co-stimulation, allows us to envision developing PVR as a dual functioning therapeutic with potential utilization in both cancer and autoimmune therapy.

Here, we constructed a modified version of PVR-Fc incorporating only its smallest functional IgV domain (vdPVR-Fc). This smaller biologic offers advantages over the classic full-length PVR-Fc in terms of production yield (data not shown). Additionally, Krippendorff et al. reported an inversed relationship between the molecular weight of proteins and their tissue biodistribution^20^. In the present study, the reduction of PVR to only its IgV domain, reduces its mass relative to the full length PVR ECD-Fc by ~70 kDa. This attribute may favor tissue penetration in some disease models and potentially enhance its therapeutic efficiency. Interestingly, the IgC domains of Ig super-family of proteins are typically important for oligomerization and molecular interactions,^21,22^ thus removing these domain(s) can render the protein unstable and reduce its binding affinity^13,23^. In this case, removing the two IgC domains from PVR did not impact its ability to bind to its cognate receptors, displaying K_d_ values within ~ 2 nM of each other, comparable to values obtained for the full-length ECD version in our study.

Of note, there is conflicting data surrounding the expected affinity of PVR for each of its cognate receptors. In the first instance, two studies reported that the affinity of PVR for TIGIT is considerably higher than its binding to CD226^10,24^. Yu et al. originally reported a 100-fold difference in affinity when comparing PVR binding to TIGIT as compared to CD226 based on radioligand binding assays on a cell line expressing these receptors^10^. In contrast, Stanietsky et al. reported similar affinities for PVR toward TIGIT and CD226, albeit in the micromolar range^6^. Complementary to the results presenting in this study, Okumura et al. recently reported that full length PVR is capable of binding to all three of its cognate receptors in the nanomolar affinity range^25^. The differences obtained across multiple studies are likely a reflection of differences in methods used to assess binding affinity (cell binding assay^10^ as compared to SPR^6^ (Table 1) or Biolayer Interferometry systems)^24,25^. The discrepancy in reported K_d_s could also be due to differences in the functional format of the cognate receptors and/or PVR used (use of recombinant pure proteins versus cell surface-expressed receptors, glycosylation patterns and oligomeric status). Nevertheless, our results indicate that it is unlikely that PVR favors binding to TIGIT over the other receptors and the signaling outcome may vary based on the interplay (signaling/crosstalk) and/or expression levels, of the cognate receptors on T-cells and NK-cells. As expected, no binding to TIGIT, CD96 and CD226 was observed for the inactive version of vdPVR-Fc incorporating two mutations Q62R and F128R (Fig. 2B). These mutations inserted in the binding motifs of PVR have previously been shown to abolish PVR binding to TIGIT and CD226 respectively^13,14^. Overall, the SPR data indicates that the PVR ECD (IgV-IgC-IgC) can be truncated to its IgV domain without a loss of binding affinities to its three cognate receptors.

From a functional perspective, T-cells are known to upregulate TIGIT upon anti-CD3/CD28 co-stimulation^5,26^. We verified the expression of TIGIT on the surface of CD4^+^ and CD8^+^ T-cells post stimulation with anti-CD3/CD28 coated beads. As expected, a net suppressive effect was observed with the addition of vdPVR-Fc based on the interplay between TIGIT and CD226, despite the co-expression of CD226 on these cells. This dampening effect was evidenced by a significant decrease in the proliferation of both CD4^+^ and CD8^+^ T-cells and a decrease in the levels of secreted IL-2 and IFNγ; known cytokine levels impacted by TIGIT agonism^26^. These findings, in combination with the lack of observed CD96 expression and the ability of vdPVR-Fc to successfully compete for TIGIT, suggest that the overall inhibitory effect observed in-vitro is TIGIT-mediated.

We subsequently tested if vdPVR-Fc displayed immunosuppressive properties in an in-vivo mouse model of plaque-like psoriasis. In this model, observable symptoms of skin inflammation are induced by the topical application of imiquimod (IMQ) cream, which activates immune responses as a TLR7 and TLR8 agonist. Full length PVR-Fc has previously shown therapeutic potential evidenced by the delay in the onset of SLE in mice^12^. However, to our knowledge, no pre-clinical work assessing the effect of in-vivo administrated PVR construct on the development of psoriasis has been attempted. One study by Fang et al. demonstrated that TIGIT expression on T cells of psoriasis patients was a predictive indicator of disease severity^15^. Additionally, T-cells isolated from psoriasis patients, treated with recombinant PVR ex-vivo, were similarly suppressed as evidenced by a decrease in CD4^+^ T-cell proliferation and reduction in pro-inflammatory cytokine output^15^. It remains unclear whether this immunosuppressive effect is mediated by TIGIT or CD96. In our study, vdPVR-Fc was demonstrated to delay the onset of psoriatic symptoms and minimized peak severity as compared to IMQ alone or in mice treated with mutant vdPVR-Fc. Based on these results, the effect of vdPVR is elicited early in the model, and while the peak severity of symptoms is reduced, the significance of the protective effect is lost. While TIGIT has been described as an important indicator of disease severity in psoriasis, our results cannot rule out that the early protective effect of vdPVR could, in part, be mediated through CD96 and that the action of vdPVR-Fc may not be fully restricted to T-cells alone. Indeed, our results from early (Day 3) assessment of immune cells within the skin indicate that vdPVR-Fc treatment results in a significant reduction in the amount of NK cells present. It is known that PVR binds to TIGIT and CD96 on the surface of NK-cells^6,7^. Furthermore, evidence is emerging supporting the involvement of NK-cells in the early development of psoriasis^27^. Likewise, we cannot rule out that the early protection elicited by vdPVR-Fc was lost due to a switch to CD226 signaling. More studies are required to determine the exact interplay between various immune cell types and the expression profiles of the PVR cognate receptors within this model before any definitive conclusions can be drawn on the mechanism of action of vdPVR-Fc.

While not explored in the context of this study, we can also envision using vdPVR-Fc as an immune co-stimulatory molecule. A recent study by Fourcade et al. demonstrated that CD226 co-stimulation using PVR-Fc synergistically with TIGIT blockade suppressed melanoma patient derived Tregs ex-vivo^28^. Furthermore, human cancers are known to differentially express PVR thus  bioavailability may be limited^29^. Based on these studies, we postulate that one of the factors limiting the success of TIGIT antagonism in the treatment of cancer could be due to the lack of synergistic CD226 co-stimulation. Future work will be aimed at investigating the potential dual-functionality of vdPVR-Fc as a co-stimulatory reagent in the presence of appropriate anti-TIGIT and anti-CD96 antagonists.

In summary, our engineered minimal IgV domain of PVR, presented as a Fc construct (vdPVR-Fc) binds tightly and equivalently to TIGIT, CD226 and CD96 (K_d_s in the low nM range), reduces the proliferation of T-cells and secretion of pro-inflammatory cytokines in-vitro, and attenuates the development of psoriasiform like symptoms in an in-vivo mouse model of psoriasis. The nanomolar affinities and functional activity observed herein for the minimal IgV-Fc construct affirms that this domain alone encodes for the functional features of PVR.

## Materials and methods

### Recombinant protein production and purification

The vdPVR-Fc construct was synthesized by linking the murine PVR IgV ECD (Accession # NP_081790.1; residues 29–147) upstream of the human IgG1 Fc region (Accession # P01857; residues 100–330) and inserted into the pcDNA3.4 TOPO mammalian expression plasmid (GeneArt; Thermo Fisher Scientific). The linker sequence IEGRMD was inserted between the PVR IgV ECD and the human IgG1 Fc domain. The mutant vdPVR-Fc construct was synthesized similarly except two-point mutations (Q62R and F128R) were inserted in the PVR IgV ECD to abolish binding to TIGIT and CD226. Both plasmids also contained a 5’ Ig-kappa leader sequence^30^ for secretion into culture media. Recombinant proteins were produced using Expi293F expression system (GeneArt; Thermo Fisher Scientific). Secreted proteins were purified using HiTrap protein A HP columns (17040301; Cytiva) and eluted with 0.1 M glycine–HCl (pH 2.7) and neutralized with 1 M Tris–HCl (pH 9.0). After purification, protein samples were desalted into PBS using PD10 columns (17085101; Cytiva) and ran through endotoxin removal columns (88274; Thermo Fisher Scientific). Finally, protein samples were verified for purity using SDS-PAGE and Western blot. The blots were probed with goat anti-human Fc fragment antibody conjugated to horseradish peroxidase (HRP) (A80-304P; Bethyl Laboratories). Protein concentrations were quantified by measuring sample absorbance at 280 nm.

### Surface plasmon resonance

Binding kinetics of vdPVR-Fc, mutant vdPVR-Fc and PVR-Fc (786608; Biolegend) proteins to mTIGIT-Fc-his (771808; Biolegend), mCD226-his (50232-M08H; SinoBiological) and mCD96-his (788806; Biolegend) were derived from single-cycle kinetic analyses by surface plasmon resonance (SPR) using a Biacore T200 (Cytiva). Briefly, anti-histidine antibodies were immobilized on a CM5 chip (29149604; Cytiva) using the His capture kit (28995056; Cytiva) following manufacturer’s protocol. The target proteins (mTIGIT-Fc-His tag, mCD226-His tag and mCD96-His tag were captured on the anti-histidine coated chip by flowing 1–5 μg/mL of protein at a flow rate of 30 µL/min. Five concentrations (1:2 serial dilutions) of vdPVR-Fc, mutant vdPVR-Fc and PVR-Fc were flown over the immobilized proteins. All proteins were diluted in HBS-EP running buffer (20 mM of HEPES pH 7.4, 150 mM NaCl, 0.005% Tween-20, 3.4 mM EDTA). The derived sensorgrams were fitted to a 1:1 Langmuir binding model and analyzed using the Biacore T200 evaluation software to calculate the on-rate (k_a_), off-rate (k_d_), and the equilibrium constant (K_D_).

### Animals

Female C57BL/6 mice, 8–12 weeks of age (Charles River Laboratories), used throughout this study were housed in a pathogen-free environment at the Sunnybrook Research Institute (SRI) Comparative Research facility. All protocols (AUP 658 and 710) were performed under the approval of the SRI Comparative Research Animal Care Committee in accordance with the rules and regulations of the Canadian Council for Animal Care. Results from animal studies are reported in accordance with the ARRIVE^31^ guidelines.

### Cell binding to mouse T-cells

Binding of vdPVR-Fc and mutant vdPVR-Fc to primary murine CD4^+^ and CD8^+^ T-cells were assessed using flow cytometry. Mouse splenocytes were treated with red blood cell lysis buffer and cultured with 2 μg/mL Concanavalin A (C5275; MilliporeSigma) for 2 days. Both constructs incorporated a human Fc domain to simplify their detection. Murine splenocytes were harvested, had their Fc receptors blocked with Mouse TruStain FcX (101319; BioLegend) and were incubated with 2.5 µg of vdPVR-Fc or mutant vdPVR-Fc for 1 h at 4 °C. Cells were then washed and PE anti-human IgG Fc (409304; Biolegend) was added to detect vdPVR-Fc and mutant PVR-Fc binding. Additionally, cells were simultaneously stained with a cocktail of Alexa Flour 700 anti-CD3 (100216; BioLegend), APC anti-CD8 (100711; BioLegend), and PE/Cy7 anti-CD4 (100422; BioLegend) for 30 min at 4°C. Cells were subsequently washed and analysed by flow cytometry (BD LSR II, Becton Dickinson; Center for Scanning Microscopy and Flow Cytometry; Sunnybrook Research Institute) with DAPI (D1306; Thermo Fisher Scientific) used to exclude dead cells. To confirm the specificity of vdPVR-Fc to TIGIT, competition cell binding experiments were performed with mouse anti-TIGIT antibody (clone 1B4; Absolute Antibody). Mouse splenocytes were incubated with vdPVR-Fc as described above. Cells were then washed and a 2-molar excess anti-TIGIT antibody was added along with the other surface markers listed above for 30 min at 4°C.

### Mouse splenocyte proliferation and cytokine production assay

Mouse splenocytes were treated with red blood cell lysis buffer, stained with 5 μM CFSE (C34554; Thermo Fisher Scientific), and cultured in RPMI-1640 media (Wisent Bio Products) supplemented with 10% FBS, penicillin (100U/mL), HEPES (20 mM) and 2-mercaptoethanol (0.05 mM). Cells were plated in round bottom 96-well plates at 3 × 10^5^ cells per well and stimulated with Dynabeads™ Mouse T-Activator CD3/CD28 (11452D; Thermo Fisher Scientific). Proteins including vdPVR-Fc and mutant vdPVR-Fc were added at 10 μg/mL. After 3 days in culture, cells were harvested, had their Fc receptors blocked with Mouse TruStain FcX (101319; BioLegend) and were stained with a cocktail of Alexa Flour 700 anti-CD3 (100216; BioLegend), APC anti-CD8 (100711; BioLegend), and PE/Cy7 anti-CD4 (100422; BioLegend). T-cell proliferation as defined by the percentage of CFSE signal distributed within dividing T-cells populations was then measured by flow cytometry. IL-2 and IFNγ secretion levels in culture were measured after 3 days using the appropriate ELISA MAX™ Deluxe Set Mouse kits (431004 and 430801; Biolegend).

TIGIT, CD226 and CD96 expression on T-cells were assessed under these culture conditions after stimulation with Dynabeads™ Mouse T-Activator CD3/CD28. Cells were stained with PE anti-TIGIT (142103; Biolegend), PerCP/Cyanine5.5 anti-CD226 (128813; Biolegend), APC anti-CD96 (131711; Biolegend) and the appropriate isotype control.

### Cytotoxicity assay

Cytotoxicity assays were performed as previously described^32,33^. In brief, 100,000 murine splenocytes were stimulated with Dynabeads™ Mouse T-Activator CD3/CD28 and cultured in the presence or absence of 10 µg/mL of either vdPVR-Fc, mutant vdPVR-Fc, human IgG1 isotype control (BE0297; BioXCell) or anti-CTLA4 (Yervoy; Bristol-Myers Squibb). Following a 4-h incubation period at 37°C, supernatants were collected, and cell cytotoxicity assayed using a lactate dehydrogenase kit (ab65393; Abcam). Percent cytotoxicity was calculated with the following formula: cytotoxicity (%) = ((Test Sample − Low Control)/(High Control − Low Control)) × 100.

### IMQ induced psoriasis model

Female C57BL/6 mice (8-week-old; Charles River Laboratories) received daily topical application on their shaved back of 62.5 mg of 5% (w/v) IMQ cream (Aldara; Valeant Pharmaceuticals) or petroleum jelly (Vaseline^®^). Groups of treated mice (n = 13) received intraperitoneal injections every other day starting from day 0 with either vdPVR-Fc (100 µg), mutant vdPVR-Fc (100 µg) or PBS (200 µL). A subset of mice in each group were sacrificed on Day 3 for skin histology and digestion while the remaining mice were treated and scored until Day 5. The severity of the psoriasis-like skin conditions was scored as follows: erythema, scaling, and thickness on a scale ranging from 0 to 4 (no symptoms, 0; mild, 1; moderate, 2; severe, 3; very severe, 4) was recorded daily in each mouse cohort. Scoring was done by 3 independent scorers who were blinded as to the treatment given. The total score was obtained by calculating the sum of the 3 index scores.

### Skin digestion and histology

Mice (n = 9) were sacrificed on Day 3 and had their skin harvested for histological and flow cytometry analysis. Murine back skin sections were digested to assess immune cell populations as previously described^34^. Briefly, precut skin sections were digested with  Collagenase P (11213857001; Sigma-Aldrich), DNAse I (9003-98-9; Worthington) and Dispase II (D4693-1G; Sigma-Aldrich) in DMEM (Wisent Bio Products) for 1 h in 37°C. Cells were then filtered through 40 μm cell strainers before being stained for flow cytometry. Next, Fc receptors on cells were blocked with Mouse TruStain FcX (101319; BioLegend) and stained with cocktail combinations of: APC/Cy7 anti-CD45 (103116; Biolegend), PE/Cy5 anti-CD3 (100310; BioLegend), APC anti-CD8, PE/Cy7 anti-CD4, Alexa Fluora 700 anti-CD11b (101222; Biolegend), FITC anti-NKp46 (137605; Biolegend), PE anti-TIGIT, PerCP Cy5.5 anti-CD226, FITC anti-F4/80 (123108; Biolegend), APC anti-Ly6C (128015; Biolegend), and PE/Cy5 anti-CD11c (117316; Biolegend).

Additionally, skin samples were sectioned and stained with H&E for measuring epidermal thickness. Histology section images were captured, and epidermal thickness was measured using S-EYE 1.4 software. The epidermal thickness was quantified by measuring thickness in at least three distinct locations on each tissue section.

### Statistical analyses and softwares

Statistical analyses were completed using GraphPad Prism 9 statistical software using one-way or two-way ANOVA to analyze mean differences between groups. P-values less than 0.05 were considered statistically significant. The mutant vdPVR-Fc ribbon model was generated using ColabFold^35^. The amino acid sequence of the mutant vdPVR-Fc construct was uploaded, and the generated ribbon model was constructed as a homodimer homo-oligomer. Flow cytometry analysis was performed using FlowJo version 10.6. (BD Life Sciences).

## Supplementary Information

Supplementary Figures.

## Data Availability

Data is available upon reasonable request from the corresponding author.
