# Supplementary material for: The IgV domain of the poliovirus receptor alone is immunosuppressive and binds to its receptors with comparable affinity
Source: Sci Rep. 2023 Mar 21;13:4609. doi: 10.1038/s41598-023-30999-w (PMC10030575; doi:10.1038/s41598-023-30999-w)
Supplement: Supplementary file 1 — Supplementary Figures. [file 41598_2023_30999_MOESM1_ESM.pdf]

The IgV domain of the poliovirus receptor alone is immunosuppressive and binds to its receptors with comparable affinity

Shrayasee Saha[1,2#], Amanda Sparkes[1#], Esther I. Matus[1,3], Peter Lee[1], Jean Gariépy[1,2,3]*

1.Physical Sciences, Sunnybrook Research Institute, Toronto, Canada

2. Department of Pharmaceutical Sciences, University of Toronto, Toronto, Canada

3.Department of Medical Biophysics, University of Toronto, Toronto, Canada

Corresponding Author: Jean Gariépy jean.gariepy@utoronto.ca Physical Sciences, Sunnybrook Research Institute, 2075 Bayview Ave., Room M7-434, Toronto, ON M4N 3M5, Canada

[#] These authors have contributed equally to this work and share first authorship

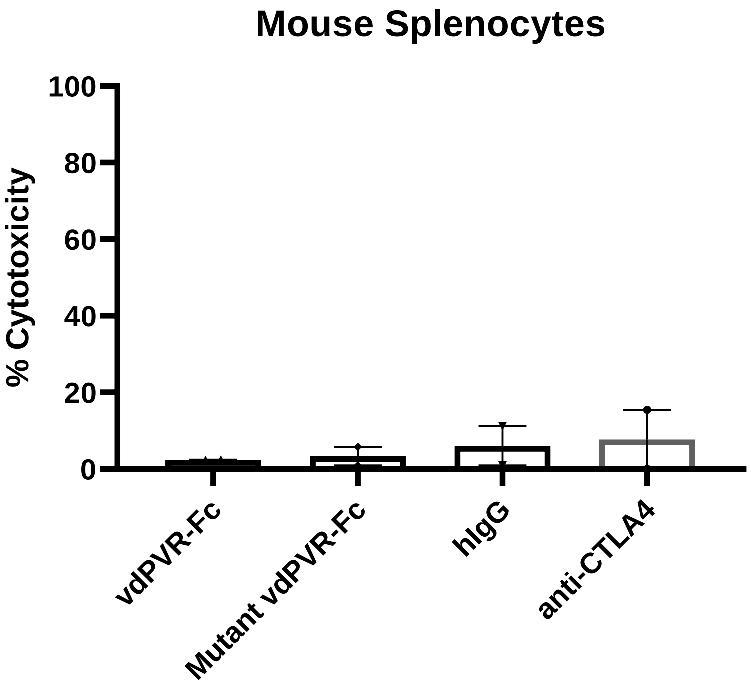

Supplementary Figure 1. Assessing the cytotoxicity of vdPVR-Fc and mutant vdPVR-Fc towards mouse splenocytes. Both vdPVR-Fc and mutant vdPVR-Fc were not cytotoxic towards mouse splenocytes. Cytotoxicity was determined by ELISA by measuring the level of lactate dehydrogenase released in cell supernatants. Cytotoxicity (%) = ((Test Sample – Low Control) / (High Control – Low Control)) ×100, where cells cultured in the absence of proteins served as a baseline negative control and signal recorded following complete cell lysis represented our positive control. Each point represents a biological replicate. (n = 2; error bars represent mean ± SEM). An isotype-matched human IgG1 and an anti-human CTLA mAb served as controls for measuring the cytotoxic effect of the Fc domain present in our constructs on mouse splenocytes.

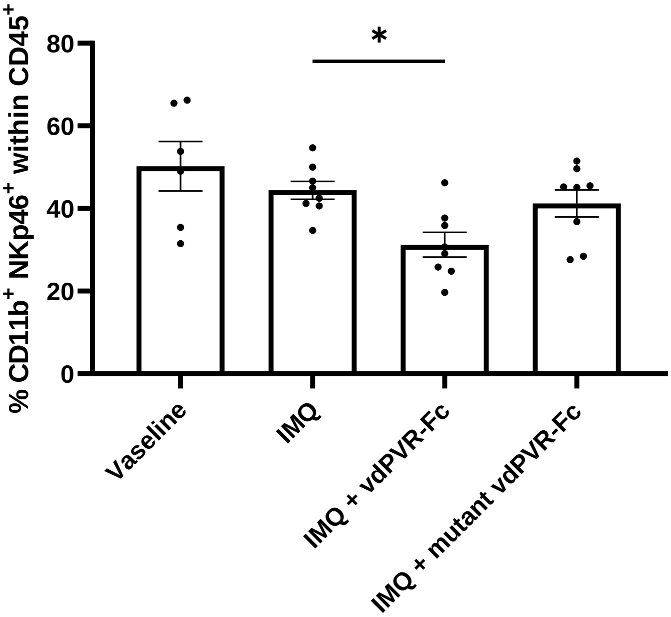

Supplementary Figure 2. vdPVR-Fc reduces CD11b$^+$ NKp46$^+$ NK-cells in the psoriatic skin. Immune cell populations in the back skin samples of mice (n= 6-8) were analyzed by flow cytometry on Day 3. Treatment with vdPVR-Fc significantly reduces CD11b$^+$ NKp46$^+$ NK-cells within the skin compared to mice receiving IMQ alone. Each point represents a biological replicate. Error bars represents mean ± SEM, * $p<0.05$ relative to IMQ alone group evaluated by one-way ANOVA.

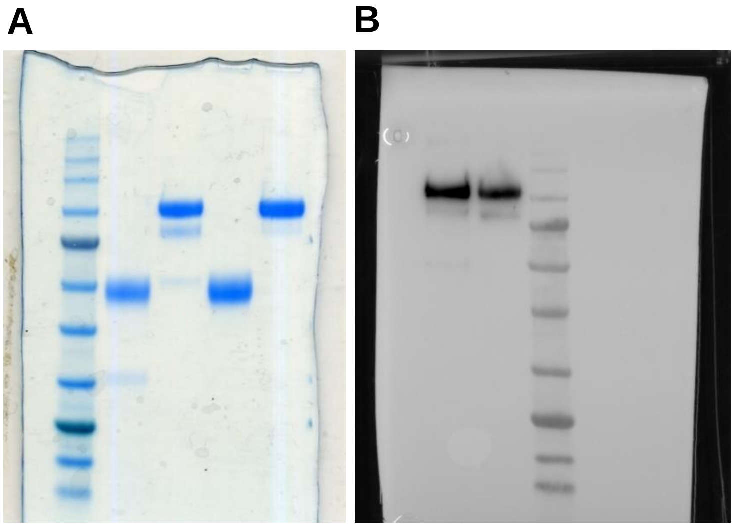

Supplementary Figure 3. Full unedited SDS gel and Western blot of vdPVR-Fc and mutant vdPVR-Fc. (A) Unedited Coomassie-stained SDS gel shown in Figure 1C. **(B)** Unedited Western blot of Figure 1C. All lanes of both the SDS gel and Western blot are shown in the manuscript figure.
